# Supplementary material for: Associations of genetic variants for educational success with risk and time preferences vary by childhood environment
Source: Commun Psychol. 2026 Feb 11;4:50. doi: 10.1038/s44271-026-00421-y (PMC13004898; doi:10.1038/s44271-026-00421-y)
Supplement: Supplementary file 2 — Supplementary Information [file 44271_2026_421_MOESM2_ESM.pdf]

Supplementary Material

For

“Associations of genetic variants for educational success with risk and time preferences vary by childhood environment”

Supplementary Table 1. Summary statistics

	Min.	Max.	Mean/Frequency	
			No disadv.	Disadv.
**Panel A – Experimental sample**				
Age	50	75	63.41	65.6
Male	0	1	0.45	0.48
PGS EA	4206.28	4279.9	4249.58	4247.75
**Risk aversion (B-EG):**				
Lottery A	0	1	0.35	0.40
Lottery B	0	1	0.20	0.18
Lottery C	0	1	0.13	0.11
Lottery D	0	1	0.09	0.09
Lottery E	0	1	0.12	0.08
Lottery F	0	1	0.11	0.13
**Risk aversion (LA):**				
Lottery A	0	1	0.43	0.49
Lottery B	0	1	0.27	0.19
Lottery C	0	1	0.09	0.07
Lottery D	0	1	0.06	0.05
Lottery E	0	1	0.05	0.07
Lottery F	0	1	0.10	0.12
**Discount rate (1-Month MPL):**				
Payoff Alternative 1	0	1	0.5	0.44
Payoff Alternative 2	0	1	0.13	0.16
Payoff Alternative 3	0	1	0.12	0.12
Payoff Alternative 4	0	1	0.03	0.05
Payoff Alternative 5	0	1	0.05	0.05
Payoff Alternative 6	0	1	0.02	0.01
Never switched	0	1	0.15	0.16
**Discount rate (2-Month MPL):**				
Payoff Alternative 1	0	1	0.34	0.31
Payoff Alternative 2	0	1	0.19	0.16
Payoff Alternative 3	0	1	0.14	0.17
Payoff Alternative 4	0	1	0.08	0.06
Payoff Alternative 5	0	1	0.07	0.09
Payoff Alternative 6	0	1	0.01	0.02
Never switched	0	1	0.17	0.18
Observations			405	219
**Panel B – Survey-based sample**				
Age	31	90	63.87	65.5
Male	0	1	0.46	0.45
PGS EA	4196.97	4296.68	4249.29	4247.37
**Planning horizon:**				
does not plan/plans day to day	0	1	0.09	0.10
the next few weeks	0	1	0.10	0.14
the next few months	0	1	0.13	0.15
the next year	0	1	0.16	0.17
the next few years	0	1	0.24	0.22
the next 5-10 years	0	1	0.20	0.17
longer than 10 years	0	1	0.08	0.06

Observations	7402	4119

Supplementary Table 2. Gene-Environment Interaction and Risk Preferences

Regression:	1	2	3	4
Dependent variable:	Risk aversion (B-EG)	Risk aversion (LA)	Risk aversion (LA-binary)	Risk aversion (LA-binary)
Estimator:	Interval	Ordered Logit	Logit	Logit
$\beta 1$ PGS EA	-0.368***	-0.350***	-0.543***	-0.483***
	[-3.000]	[-4.072]	[-4.677]	[-3.960]
$\beta 2$ PGS EA × Disadv.	0.799***	0.715***	0.706***	0.575**
	[3.609]	[4.135]	[3.446]	[2.524]
$\beta 1 + \beta 2$	0.431**	0.365**	0.164	0.092
	[2.334]	[2.442]	[0.968]	[0.478]
Controls	Yes	Yes	Yes	Yes
Observations	624	624	624	624

Note. The dependent variable in: Regression 1 uses the upper and lower bounds of participants' implied risk-aversion coefficients from the B–EG task. Regression 2 uses a 6-point scale from the adapted B–EG task that includes losses, ranging from 1 = most risk-seeking (Lottery F) to 6 = most risk-averse (Lottery A). Regressions 3 and 4 use a dichotomous indicator equal to 1 for loss-free lotteries (Lotteries A–C) and 0 for lotteries that include a loss (Lotteries D–F) from the adapted B–EG task. Polygenic score for educational attainment (PGS EA) is standardized. Unadjusted coefficients; z-statistics using robust standard errors in brackets. Controls include age (in linear and quadratic form), sex, and the first 10 principal components of respondents' SNPs. In Regression 4, we also include a series of dummy variables representing respondents' choices in the B–EG task. In all regressions, all parameters are allowed to differ across groups ('no disadvantage' and 'disadvantage').
* p<0.10, ** p<0.05, *** p<0.01.

Supplementary Table 3. Gene-Environment Interaction and Time Preferences

Regression:	1	2	3	4	5
Dependent variable:	Discount rate (1-Month MPL)	Discount rate (2-Month MPL)	Discount rate (2-Month MPL)	Planning horizon	Planning horizon (binary)
Estimator:	Interval	Interval	Interval	Ordered Logit	Logit
$\beta 1$ PGS EA	-0.021**	-0.011***	-0.006**	0.199***	0.196***
	[-2.479]	[-3.690]	[-2.412]	[7.913]	[7.183]
$\beta 2$ PGS EA × Disadv.	-0.007	0.007	0.009**	-0.131***	-0.146***
	[-0.477]	[1.351]	[2.481]	[-3.263]	[-3.249]
$\beta 1 + \beta 2$	-0.027**	-0.004	0.004	0.068**	0.049
	[-2.419]	[-1.039]	[1.293]	[2.173]	[1.368]
Controls	Yes	Yes	Yes	Yes	Yes
Observations	624	624	624	11521	11521

Note. The dependent variable in: Regression 1 uses the upper and lower bounds of participants' implied discount rate from the 1-month multiple price list (MPL) task. Regressions 2 and 3 use participants' implied discount rates from the 2-month MPL task. Regression 4 uses a 7-point planning horizon scale ranging from 0 ("does not plan/plans day-to-day") to 6 ("longer than 10 years"). Regression 5 uses a dichotomous planning horizon indicator equal to 1 if the respondent reports planning beyond the next year, and 0 otherwise. Polygenic score for educational attainment (PGS EA) is standardized. Unadjusted coefficients; z-statistics using robust standard errors in brackets (cluster-robust standard errors in Regressions 4 and 5). Controls include age (in linear and quadratic form), sex, and the first 10 principal components of respondents' SNPs. In Regressions 1-3 we also include a dichotomous control variable that equals 1 if the subject made an inconsistent choice in the MPL task and 0 otherwise. In Regression 3, we also include a series of dummy variables representing respondents' choice from the 1-month MPL task. In all regressions, all parameters are allowed to differ across groups ('no disadvantage' and 'disadvantage').

* p<0.10, ** p<0.05, *** p<0.01.

Supplementary Table 4. Gene-Environment Interaction and Risk Preferences – Valid Responses to all four Childhood Disadvantage Dimensions

Regression:	1	2	3	4
Dependent variable:	Risk aversion (B-EG)	Risk aversion (LA)	Risk aversion (LA-binary)	Risk aversion (LA-binary)
Estimator:	Interval	Ordered Logit	Logit	Logit
$\beta 1$ PGS EA	-0.430***	-0.407***	-0.605***	-0.551***
	[-3.012]	[-3.669]	[-4.163]	[-3.390]
$\beta 2$ PGS EA × Disadv.	0.912***	0.771***	0.689***	0.531*
	[3.717]	[3.648]	[2.740]	[1.841]
$\beta 1 + \beta 2$	0.482**	0.364**	0.084	-0.021
	[2.400]	[2.043]	[0.410]	[-0.086]
Controls	Yes	Yes	Yes	Yes
Observations	432	432	432	432

Note. The dependent variable in: Regression 1 uses the upper and lower bounds of participants' implied risk-aversion coefficients from the B–EG task. Regression 2 uses a 6-point scale from the adapted B–EG task that includes losses, ranging from 1 = most risk-seeking (Lottery F) to 6 = most risk-averse (Lottery A). Regressions 3 and 4 use a dichotomous indicator equal to 1 for loss-free lotteries (Lotteries A–C) and 0 for lotteries that include a loss (Lotteries D–F) from the adapted B–EG task. Polygenic score for educational attainment (PGS EA) is standardized. Unadjusted coefficients; z-statistics using robust standard errors in brackets. Controls include age (in linear and quadratic form), sex, and the first 10 principal components of respondents' SNPs. In Regression 4, we also include a series of dummy variables representing respondents' choice in the B-EG task. In all regressions, all parameters are allowed to differ across groups ('no disadvantage' and 'disadvantage').
* p<0.10, ** p<0.05, *** p<0.01.

Supplementary Table 5. Gene-Environment Interaction and Time Preferences – Valid Responses to all four Childhood Disadvantage Dimensions

Regression:	1	2	3	4	5
Dependent variable:	Discount rate (1-Month MPL)	Discount rate (2-Month MPL)	Discount rate (2-Month MPL)	Planning horizon	Planning horizon (binary)
Estimator:	Interval	Interval	Interval	Ordered Logit	Logit
$\beta 1$ PGS EA	-0.017*	-0.012***	-0.007***	0.241***	0.248***
	[-1.737]	[-3.283]	[-2.712]	[7.065]	[6.472]
$\beta 2$ PGS EA × Disadv.	-0.006	0.010*	0.012***	-0.174***	-0.189***
	[-0.370]	[1.705]	[2.746]	[-3.390]	[-3.283]
$\beta 1 + \beta 2$	-0.023*	-0.002	0.005	0.068*	0.059
	[-1.718]	[-0.324]	[1.458]	[1.763]	[1.390]
Controls	Yes	Yes	Yes	Yes	Yes
Observations	432	432	432	6770	6770

Note. The dependent variable in: Regression 1 uses the upper and lower bounds of participants' implied discount rate from the 1-month multiple price list (MPL) task. Regressions 2 and 3 use participants' implied discount rates from the 2-month MPL task. Regression 4 uses a 7-point planning horizon scale ranging from 0 ("does not plan/plans day-to-day") to 6 ("longer than 10 years"). Regression 5 uses a dichotomous planning horizon indicator equal to 1 if the respondent reports planning beyond the next year, and 0 otherwise. Polygenic score for educational attainment (PGS EA) is standardized. Unadjusted coefficients; z-statistics using robust standard errors in brackets (cluster-robust standard errors in Regressions 4 and 5). Controls include age (in linear and quadratic form), sex, and the first 10 principal components of respondents' SNPs. In Regressions 1-3 we also include a dichotomous control variable that equals 1 if the subject made an inconsistent choice in the MPL task and 0 otherwise. In Regression 3, we also include a series of dummy variables representing respondents' choice from the 1-month MPL task. In all regressions, all parameters are allowed to differ across groups ('no disadvantage' and 'disadvantage').

* p<0.10, ** p<0.05, *** p<0.01.

Supplementary Table 6. Gene-Environment Interaction and Risk Preferences – Missingness

Regression:	1	2	3	4
Dependent variable:	Risk aversion (B-EG)	Risk aversion (LA)	Risk aversion (LA-binary)	Risk aversion (LA-binary)
Estimator:	Interval	Ordered Logit	Logit	Logit
$\beta 1$ PGS EA	-0.373***	-0.352***	-0.540***	-0.471***
	[-3.040]	[-4.072]	[-4.648]	[-3.847]
$\beta 2$ PGS EA × Disadv.	0.800***	0.717***	0.704***	0.565**
	[3.620]	[4.098]	[3.443]	[2.475]
$\beta 1 + \beta 2$	0.428**	0.365**	0.164	0.094
	[2.320]	[2.408]	[0.975]	[0.487]
Controls	Yes	Yes	Yes	Yes
Missingness indicator	Yes	Yes	Yes	Yes
Observations	624	624	624	624

Note. The dependent variable in: Regression 1 uses the upper and lower bounds of participants' implied risk-aversion coefficients from the B–EG task. Regression 2 uses a 6-point scale from the adapted B–EG task that includes losses, ranging from 1 = most risk-seeking (Lottery F) to 6 = most risk-averse (Lottery A). Regressions 3 and 4 use a dichotomous indicator equal to 1 for loss-free lotteries (Lotteries A–C) and 0 for lotteries that include a loss (Lotteries D–F) from the adapted B–EG task. Polygenic score for educational attainment (PGS EA) is standardized. Unadjusted coefficients; z-statistics using robust standard errors in brackets. Controls include age (in linear and quadratic form), sex, and the first 10 principal components of respondents' SNPs. Missingness indicator is a dichotomous variable that takes on the value of 1 if the respondent did not have valid responses to all four disadvantage dimensions and 0 otherwise. In Regression 4, we also include a series of dummy variables representing respondents' choice in the B-EG task. In all regressions, all parameters are allowed to differ across groups ('no disadvantage' and 'disadvantage').
* p<0.10, ** p<0.05, *** p<0.01.

Supplementary Table 7. Gene-Environment Interaction and Time Preferences – Missingness

Regression:	1	2	3	4	5
Dependent variable:	Discount rate (1-Month MPL)	Discount rate (2-Month MPL)	Discount rate (2-Month MPL)	Planning horizon	Planning horizon (binary)
Estimator:	Interval	Interval	Interval	Ordered Logit	Logit
$\beta 1$ PGS EA	-0.021**	-0.011***	-0.005**	0.184***	0.179***
	[-2.505]	[-3.672]	[-2.372]	[7.275]	[6.531]
$\beta 2$ PGS EA × Disadv.	-0.006	0.007	0.010**	-0.122***	-0.135***
	[-0.431]	[1.376]	[2.508]	[-3.037]	[-2.982]
$\beta 1 + \beta 2$	-0.027**	-0.004	0.004	0.061*	0.044
	[-2.362]	[-0.987]	[1.364]	[1.942]	[1.221]
Controls	Yes	Yes	Yes	Yes	Yes
Missingness indicator	Yes	Yes	Yes	Yes	Yes
Observations	624	624	624	11521	11521

Note. The dependent variable in: Regression 1 uses the upper and lower bounds of participants' implied discount rate from the 1-month multiple price list (MPL) task. Regressions 2 and 3 use participants' implied discount rates from the 2-month MPL task. Regression 4 uses a 7-point planning horizon scale ranging from 0 ("does not plan/plans day-to-day") to 6 ("longer than 10 years"). Regression 5 uses a dichotomous planning horizon indicator equal to 1 if the respondent reports planning beyond the next year, and 0 otherwise. Polygenic score for educational attainment (PGS EA) is standardized. Unadjusted coefficients; z-statistics using robust standard errors in brackets (cluster-robust standard errors in Regressions 4 and 5). Controls include age (in linear and quadratic form), sex, and the first 10 principal components of respondents' SNPs. In Regressions 1-3 we also include a dichotomous control variable that equals 1 if the subject made an inconsistent choice in the MPL task and 0 otherwise. In Regression 3, we also include a series of dummy variables representing respondents' choice from the 1-month MPL task. Missingness indicator is a dichotomous variable that takes on the value of 1 if the respondent did not have valid responses to all four disadvantage dimensions and 0 otherwise. In all regressions, all parameters are allowed to differ across groups ('no disadvantage' and 'disadvantage').
* p<0.10, ** p<0.05, *** p<0.01.

Supplementary Table 8. Gene-Environment Interaction and Risk Preferences – Separate Dimensions of Childhood Disadvantage

Regression:	1	2	3	4
Dependent variable:	Risk aversion (B-EG)	Risk aversion (LA)	Risk aversion (LA-binary)	Risk aversion (LA-binary)
Estimator:	Interval	Ordered Logit	Logit	Logit
**Panel A.** Human capital disadvantage - neither parent with education above the compulsory level				
$\beta 1$ PGS EA	-0.284*	-0.260**	-0.436***	-0.441***
	[-1.809]	[-2.362]	[-3.019]	[-2.862]
$\beta 2$ PGS EA × Disadv.	0.292	0.229	0.260	0.266
	[1.363]	[1.500]	[1.321]	[1.227]
$\beta 1 + \beta 2$	0.008	-0.031	-0.175	-0.175
	[0.055]	[-0.297]	[-1.304]	[-1.146]
**Panel B.** Resource disadvantage - main caregiver low status occupation				
$\beta 1$ PGS EA	-0.213*	-0.248***	-0.354***	-0.335***
	[-1.786]	[-3.080]	[-3.318]	[-2.940]
$\beta 2$ PGS EA × Disadv.	0.334	0.515***	0.334	0.382
	[1.294]	[2.805]	[1.470]	[1.449]
$\beta 1 + \beta 2$	0.121	0.267	-0.020	0.048
	[0.528]	[1.621]	[-0.101]	[0.201]
**Panel C.** Instability disadvantage - parents being permanently separated or divorced				
$\beta 1$ PGS EA	-0.231**	-0.209**	-0.373***	-0.318***
	[-1.993]	[-2.523]	[-3.454]	[-2.785]
$\beta 2$ PGS EA × Disadv.	0.735**	0.415*	0.657**	0.442
	[2.391]	[1.750]	[2.146]	[1.168]
$\beta 1 + \beta 2$	0.504*	0.206	0.284	0.124
	[1.771]	[0.929]	[0.992]	[0.345]
**Panel D.** Resource disadvantage - childhood home no central heating or hot water				
$\beta 1$ PGS EA	-0.222*	-0.228***	-0.448***	-0.415***
	[-1.863]	[-2.713]	[-4.090]	[-3.634]
$\beta 2$ PGS EA × Disadv.	0.465*	0.345*	0.517**	0.467*
	[1.703]	[1.796]	[2.193]	[1.903]
$\beta 1 + \beta 2$	0.243***	0.117***	0.069***	0.052***
	[0.989]	[0.674]	[0.331]	[0.238]
Controls	Yes	Yes	Yes	Yes
Observations	624	624	624	624

Note. The dependent variable in: Regression 1 uses the upper and lower bounds of participants' implied risk-aversion coefficients from the B–EG task. Regression 2 uses a 6-point scale from the adapted B–EG task that includes losses, ranging from 1 = most risk-seeking (Lottery F) to 6 = most risk-averse (Lottery A). Regressions 3 and 4 use a dichotomous indicator equal to 1

for loss-free lotteries (Lotteries A–C) and 0 for lotteries that include a loss (Lotteries D–F) from the adapted B–EG task. Polygenic score for educational attainment (PGS EA) is standardized. Unadjusted coefficients; *z*-statistics using robust standard errors in brackets. Controls include age (in linear and quadratic form), sex, and the first 10 principal components of respondents' SNPs. In Regression 4, we also include a series of dummy variables representing respondents' choice in the B-EG task. In all regressions, all parameters are allowed to differ across groups ('no disadvantage' and 'disadvantage').
* p<0.10, ** p<0.05, *** p<0.01.

Supplementary Table 9. Gene-Environment Interaction and Time Preferences – Separate Dimensions of Childhood Disadvantage

Regression:	1	2	3	4	5
Dependent variable:	Discount rate (1-Month MPL)	Discount rate (2-Month MPL)	Discount rate (2-Month MPL)	Planning horizon	Planning horizon (binary)
Estimator:	Interval	Interval	Interval	Ordered Logit	Logit
**Panel A.** Human capital disadvantage - neither parent with education above the compulsory level					
$\beta1$ PGS EA	-0.026**	-0.012***	-0.004	0.188***	0.180***
	[-2.569]	[-3.047]	[-1.290]	[7.095]	[6.250]
$\beta2$ PGS EA × Disadv.	0.004	0.004	0.001	-0.086**	-0.084*
	[0.320]	[0.745]	[0.314]	[-2.182]	[-1.913]
$\beta1 + \beta2$	-0.022**	-0.008**	-0.002	0.102***	0.096***
	[-2.445]	[-2.313]	[-0.948]	[3.443]	[2.885]
**Panel B.** Resource disadvantage - main caregiver low status occupation					
$\beta1$ PGS EA	-0.025***	-0.011***	-0.004*	0.174***	0.184***
	[-3.287]	[-3.773]	[-1.660]	[7.082]	[6.932]
$\beta2$ PGS EA × Disadv.	0.006	0.008	0.005	-0.086**	-0.140***
	[0.431]	[1.406]	[1.292]	[-2.118]	[-3.031]
$\beta1 + \beta2$	-0.019	-0.003	0.001	0.088***	0.045
	[-1.507]	[-0.700]	[0.367]	[2.707]	[1.182]
**Panel C.** Instability disadvantage - parents being permanently separated or divorced					
$\beta1$ PGS EA	-0.020***	-0.009***	-0.004**	0.164***	0.146***
	[-2.887]	[-3.500]	[-2.023]	[7.630]	[6.210]
$\beta2$ PGS EA × Disadv.	-0.029	0.001	0.012**	-0.064	0.009
	[-1.388]	[0.161]	[2.070]	[-1.164]	[0.142]
$\beta1 + \beta2$	-0.049**	-0.008	0.008	0.101**	0.155***
	[-2.508]	[-1.054]	[1.500]	[1.993]	[2.740]

**Panel D.** Resource disadvantage - childhood home no central heating or hot water					
$\beta1$ PGS EA	-0.021***	-0.008***	-0.002	0.185***	0.177***
	[-2.832]	[-2.619]	[-0.903]	[7.810]	[6.878]
$\beta2$ PGS EA × Disadv.	-0.017	-0.008	-0.001	-0.100**	-0.102**
	[-1.049]	[-1.361]	[-0.227]	[-2.353]	[-2.149]
$\beta1 + \beta2$	-0.038***	-0.015***	-0.003	0.085**	0.075*
	[-2.630]	[-3.095]	[-0.852]	[2.389]	[1.885]
Controls	Yes	Yes	Yes	Yes	Yes
Observations	624	624	624	624	624

Note. The dependent variable in: Regression 1 uses the upper and lower bounds of participants' implied discount rate from the 1-month multiple price list (MPL) task. Regressions 2 and 3 use participants' implied discount rates from the 2-month MPL task. Regression 4 uses a 7-point planning horizon scale ranging from 0 ("does not plan/plans day-to-day") to 6 ("longer than 10 years"). Regression 5 uses a dichotomous planning horizon indicator equal to 1 if the respondent reports planning beyond the next year, and 0 otherwise. Polygenic score for educational attainment (PGS EA) is standardized. Unadjusted coefficients; z-statistics using robust standard errors in brackets (cluster-robust standard errors in Regressions 4 and 5). Controls include age (in linear and quadratic form), sex, and the first 10 principal components of respondents' SNPs. In Regressions 1-3 we also include a dichotomous control variable that equals 1 if the subject made an inconsistent choice in the MPL task and 0 otherwise. In Regression 3, we also include a series of dummy variables representing respondents' choice from the 1-month MPL task. In all regressions, all parameters are allowed to differ across groups ('no disadvantage' and 'disadvantage').
* p<0.10, ** p<0.05, *** p<0.01.

Supplementary Table 10. Gene-Environment Interaction and Risk Preferences – Continuous Childhood Disadvantage Measure

Regression:	1	2	3	4
Dependent variable:	Risk aversion (B-EG)	Risk aversion (LA)	Risk aversion (LA-binary)	Risk aversion (LA-binary)
Estimator:	Interval	Ordered Logit	Logit	Logit
$\beta 1$ PGS EA	-0.644***	-0.562***	-0.730***	-0.632***
	[-3.815]	[-4.245]	[-4.499]	[-3.493]
$\beta 2$ PGS EA × Disadv.	0.429***	0.360***	0.337***	0.268**
	[3.613]	[3.828]	[3.175]	[2.165]
Controls	Yes	Yes	Yes	Yes
Observations	624	624	624	624

Note. The dependent variable in: Regression 1 uses the upper and lower bounds of participants' implied risk-aversion coefficients from the B–EG task. Regression 2 uses a 6-point scale from the adapted B–EG task that includes losses, ranging from 1 = most risk-seeking (Lottery F) to 6 = most risk-averse (Lottery A). Regressions 3 and 4 use a dichotomous indicator equal to 1 for loss-free lotteries (Lotteries A–C) and 0 for lotteries that include a loss (Lotteries D–F) from the adapted B–EG task. Polygenic score for educational attainment (PGS EA) is standardized. Unadjusted coefficients; z-statistics using robust standard errors in brackets. Controls include age (in linear and quadratic form), sex, and the first 10 principal components of respondents' SNPs. In Regression 4, we also include a series of dummy variables representing respondents' choice in the B-EG task. In all regressions, all parameters are allowed to differ across groups ('no disadvantage' and 'disadvantage').
* p<0.10, ** p<0.05, *** p<0.01.

Supplementary Table 11. Gene-Environment Interaction and Time Preferences – Continuous Childhood Disadvantage Measure

Regression:	1	2	3	4	5
Dependent variable:	Discount rate (1-Month MPL)	Discount rate (2-Month MPL)	Discount rate (2-Month MPL)	Planning horizon	Planning horizon (binary)
Estimator:	Interval	Interval	Interval	Ordered Logit	Logit
$\beta 1$ PGS EA	-0.022*	-0.013***	-0.007**	0.214***	0.211***
	[-1.947]	[-3.031]	[-2.135]	[6.919]	[6.248]
$\beta 2$ PGS EA × Disadv.	-0.001	0.003	0.004*	-0.057***	-0.061***
	[-0.187]	[1.075]	[1.737]	[-3.019]	[-2.889]
Controls	Yes	Yes	Yes	Yes	Yes
Observations	624	624	624	11521	11521

Note. The dependent variable in: Regression 1 uses the upper and lower bounds of participants' implied discount rate from the 1-month multiple price list (MPL) task. Regressions 2 and 3 use participants' implied discount rates from the 2-month MPL task. Regression 4 uses a 7-point planning horizon scale ranging from 0 ("does not plan/plans day-to-day") to 6 ("longer than 10 years"). Regression 5 uses a dichotomous planning horizon indicator equal to 1 if the respondent reports planning beyond the next year, and 0 otherwise. Polygenic score for educational attainment (PGS EA) is standardized. Unadjusted coefficients; z-statistics using robust standard errors in brackets (cluster-robust standard errors in Regressions 4 and 5). Controls include age (in linear and quadratic form), sex, and the first 10 principal components of respondents' SNPs. In Regressions 1-3 we also include a dichotomous control variable that equals 1 if the subject made an inconsistent choice in the MPL task and 0 otherwise. In Regression 3, we also include a series of dummy variables representing respondents' choice from the 1-month MPL task. In all regressions, all parameters are allowed to differ across groups ('no disadvantage' and 'disadvantage').

* $p<0.10$, ** $p<0.05$, *** $p<0.01$.

Supplementary Table 12. Gene-Environment Interaction and Risk Preferences – Parental Bonding Instrument

Regression:	1	2	3	4
Dependent variable:	Risk aversion (B-EG)	Risk aversion (LA)	Risk aversion (LA-binary)	Risk aversion (LA-binary)
Estimator:	Interval	Ordered Logit	Logit	Logit
$\beta 1$ PGS EA	-0.373***	-0.354***	-0.541***	-0.477***
	[-3.070]	[-4.116]	[-4.684]	[-3.914]
$\beta 2$ PGS EA × Disadv.	0.796***	0.725***	0.716***	0.586**
	[3.625]	[4.215]	[3.517]	[2.557]
$\beta 1 + \beta 2$	0.423**	0.370**	0.175	0.109
	[2.310]	[2.501]	[1.044]	[0.563]
Controls	Yes	Yes	Yes	Yes
Parental Bonding Instrument	Yes	Yes	Yes	Yes
Observations	624	624	624	624

Note. The dependent variable in: Regression 1 uses the upper and lower bounds of participants' implied risk-aversion coefficients from the B–EG task. Regression 2 uses a 6-point scale from the adapted B–EG task that includes losses, ranging from 1 = most risk-seeking (Lottery F) to 6 = most risk-averse (Lottery A). Regressions 3 and 4 use a dichotomous indicator equal to 1 for loss-free lotteries (Lotteries A–C) and 0 for lotteries that include a loss (Lotteries D–F) from the adapted B–EG task. Polygenic score for educational attainment (PGS EA) is standardized. Unadjusted coefficients; z-statistics using robust standard errors in brackets. Controls include age (in linear and quadratic form), sex, and the first 10 principal components of respondents' SNPs. In Regression 4, we also include a series of dummy variables representing respondents' choice in the B-EG task. Parental Bonding Instrument is a continuous measure of respondents' retrospective experiences of their parents' parenting style before age 16. In all regressions, all parameters are allowed to differ across groups ('no disadvantage' and 'disadvantage').

* p<0.10, ** p<0.05, *** p<0.01.

Supplementary Table 13. Gene-Environment Interaction and Time Preferences – Parental Bonding Instrument

Regression:	1	2	3	4	5
Dependent variable:	Discount rate (1-Month MPL)	Discount rate (2-Month MPL)	Discount rate (2-Month MPL)	Planning horizon	Planning horizon (binary)
Estimator:	Interval	Interval	Interval	Ordered Logit	Logit
$\beta 1$ PGS EA	-0.021**	-0.011***	-0.005**	0.185***	0.181***
	[-2.473]	[-3.618]	[-2.335]	[7.340]	[6.581]
$\beta 2$ PGS EA × Disadv.	-0.007	0.007	0.010**	-0.121***	-0.136***
	[-0.504]	[1.348]	[2.520]	[-2.996]	[-2.997]
$\beta 1 + \beta 2$	-0.028**	-0.004	0.004	0.064**	0.045
	[-2.427]	[-1.000]	[1.409]	[2.040]	[1.241]
Controls	Yes	Yes	Yes	Yes	Yes
Parental Bonding Instrument	Yes	Yes	Yes	Yes	Yes
Observations	624	624	624	11521	11521

Note. The dependent variable in: Regression 1 uses the upper and lower bounds of participants' implied discount rate from the 1-month multiple price list (MPL) task. Regressions 2 and 3 use participants' implied discount rates from the 2-month MPL task. Regression 4 uses a 7-point planning horizon scale ranging from 0 ("does not plan/plans day-to-day") to 6 ("longer than 10 years"). Regression 5 uses a dichotomous planning horizon indicator equal to 1 if the respondent reports planning beyond the next year, and 0 otherwise. Polygenic score for educational attainment (PGS EA) is standardized. Unadjusted coefficients; z-statistics using robust standard errors in brackets (cluster-robust standard errors in Regressions 4 and 5). Controls include age (in linear and quadratic form), sex, and the first 10 principal components of respondents' SNPs. In Regressions 1-3 we also include a dichotomous control variable that equals 1 if the subject made an inconsistent choice in the MPL task and 0 otherwise. In Regression 3, we also include a series of dummy variables representing respondents' choice from the 1-month MPL task. Parental Bonding Instrument is a continuous measure of respondents' retrospective experiences of their parents' parenting style before age 16. In all regressions, all parameters are allowed to differ across groups ('no disadvantage' and 'disadvantage').

* p<0.10, ** p<0.05, *** p<0.01.

Supplementary Table 14. Gene-Environment Interaction and Risk Preferences – PGS Big 5

Regression:	1	2	3	4
Dependent variable:	Risk aversion (B-EG)	Risk aversion (LA)	Risk aversion (LA-binary)	Risk aversion (LA-binary)
Estimator:	Interval	Ordered Logit	Logit	Logit
$\beta 1$ PGS EA	-0.394***	-0.354***	-0.573***	-0.532***
	[-3.215]	[-4.064]	[-4.735]	[-4.047]
$\beta 2$ PGS EA × Disadv.	0.797***	0.740***	0.657***	0.512**
	[3.473]	[4.057]	[3.064]	[2.036]
$\beta 1 + \beta 2$	0.404**	0.385**	0.084	-0.020
	[2.075]	[2.423]	[0.476]	[-0.093]
Controls	Yes	Yes	Yes	Yes
PGS Big 5	Yes	Yes	Yes	Yes
Observations	624	624	624	624

Note. The dependent variable in: Regression 1 uses the upper and lower bounds of participants' implied risk-aversion coefficients from the B–EG task. Regression 2 uses a 6-point scale from the adapted B–EG task that includes losses, ranging from 1 = most risk-seeking (Lottery F) to 6 = most risk-averse (Lottery A). Regressions 3 and 4 use a dichotomous indicator equal to 1 for loss-free lotteries (Lotteries A–C) and 0 for lotteries that include a loss (Lotteries D–F) from the adapted B–EG task. Polygenic score for educational attainment (PGS EA) is standardized. Unadjusted coefficients; z-statistics using robust standard errors in brackets. Controls include age (in linear and quadratic form), sex, and the first 10 principal components of respondents' SNPs. In Regression 4, we also include a series of dummy variables representing respondents' choice in the B-EG task. PGS Big 5 includes the polygenic scores for the Big Five personality traits. The GWAS meta-analyses for Extraversion was conducted by the Genetics of Personality Consortium (GPC; van den Berg et al., 2018); the polygenic scores for Agreeableness, Openness to Experience and Conscientiousness were based on the GWAS meta-analysis from de Moor et al (2012); and the polygenic score for Neuroticism was calculated based on the GWAS summary statistics that collated results from the Genetics of Personality Consortium (GPC) and results from a new analysis of UKB data cohort (Okbay et al., 2016). In all regressions, all parameters are allowed to differ across groups ('no disadvantage' and 'disadvantage').

* p<0.10, ** p<0.05, *** p<0.01.

Supplementary Table 15. Gene-Environment Interaction and Time Preferences – PGS Big 5

Regression:	1	2	3	4	5
Dependent variable:	Discount rate (1-Month MPL)	Discount rate (2-Month MPL)	Discount rate (2-Month MPL)	Planning horizon	Planning horizon (binary)
Estimator:	Interval	Interval	Interval	Ordered Logit	Logit
$\beta 1$ PGS EA	-0.022***	-0.012***	-0.006**	0.199***	0.198***
	[-2.711]	[-3.824]	[-2.452]	[7.857]	[7.226]
$\beta 2$ PGS EA × Disadv.	-0.004	0.008	0.009**	-0.129***	-0.146***
	[-0.253]	[1.478]	[2.448]	[-3.178]	[-3.195]
$\beta 1 + \beta 2$	-0.026**	-0.004	0.004	0.070**	0.052
	[-2.309]	[-0.953]	[1.203]	[2.207]	[1.434]
Controls	Yes	Yes	Yes	Yes	Yes
PGS Big 5	Yes	Yes	Yes	Yes	Yes
Observations	624	624	624	11521	11521

Note. The dependent variable in: Regression 1 uses the upper and lower bounds of participants' implied discount rate from the 1-month multiple price list (MPL) task. Regressions 2 and 3 use participants' implied discount rates from the 2-month MPL task. Regression 4 uses a 7-point planning horizon scale ranging from 0 ("does not plan/plans day-to-day") to 6 ("longer than 10 years"). Regression 5 uses a dichotomous planning horizon indicator equal to 1 if the respondent reports planning beyond the next year, and 0 otherwise. Polygenic score for educational attainment (PGS EA) is standardized. Unadjusted coefficients; z-statistics using robust standard errors in brackets (cluster-robust standard errors in Regressions 4 and 5). Controls include age (in linear and quadratic form), sex, and the first 10 principal components of respondents' SNPs. In Regressions 1-3 we also include a dichotomous control variable that equals 1 if the subject made an inconsistent choice in the MPL task and 0 otherwise. In Regression 3, we also include a series of dummy variables representing respondents' choice from the 1-month MPL task. PGS Big 5 includes the polygenic scores for the Big Five personality traits. The GWAS meta-analyses for Extraversion was conducted by the Genetics of Personality Consortium (GPC; van den Berg et al., 2018); the polygenic scores for Agreeableness, Openness to Experience and Conscientiousness were based on the GWAS meta-analysis from de Moor et al (2012); and the polygenic score for Neuroticism was calculated based on the GWAS summary statistics that collated results from the Genetics of Personality Consortium (GPC) and results from a new analysis of UKB data cohort (Okbay et al., 2016). In all regressions, all parameters are allowed to differ across groups ('no disadvantage' and 'disadvantage').
* p<0.10, ** p<0.05, *** p<0.01.

Supplementary Table 16. Correlation of Polygenic Scores – Experimental Sample

Variables	(1)	(2)	(3)	(4)	(5)
(1) PGS EA					
(2) PGS Agreeableness	0.135***				
(3) PGS Conscientiousness	0.010	-0.030			
(4) PGS Extraversion	0.010	0.115***	0.040		
(5) PGS Neuroticism	-0.088**	-0.181***	0.000	-0.304***	
(6) PGS Openness	0.050	0.020	-0.150***	0.040	0.030

Note. Sample of 624 individuals.

Supplementary Table 17. Correlation of Polygenic Scores – Survey Sample

Variables	(1)	(2)	(3)	(4)	(5)
(1) PGS EA					
(2) PGS Agreeableness	0.116***				
(3) PGS Conscientiousness	-0.010	-0.034***			
(4) PGS Extraversion	-0.010	0.138***	-0.000		
(5) PGS Neuroticism	-0.102***	-0.177***	0.000	-0.265***	
(6) PGS Openness	0.010	0.020**	-0.165***	0.010	0.010

Note. Sample of 11521 observations from 5881 individuals.

Supplementary Table 18. Gene-Environment Interaction and Risk Preferences – PGS IQ

Regression:	1	2	3	4
Dependent variable:	Risk aversion (B-EG)	Risk aversion (LA)	Risk aversion (LA-binary)	Risk aversion (LA-binary)
Estimator:	Interval	Ordered Logit	Logit	Logit
$\beta 1$ PGS IQ	-0.290**	-0.231***	-0.203*	-0.133
	[-2.341]	[-2.778]	[-1.807]	[-1.044]
$\beta 2$ PGS IQ × Disadv.	0.533**	0.379**	0.370*	0.245
	[2.385]	[2.145]	[1.811]	[1.155]
$\beta 1 + \beta 2$	0.243	0.148	0.168	0.112
	[1.308]	[0.952]	[0.981]	[0.661]
Controls	Yes	Yes	Yes	Yes
Observations	624	624	624	624

Note. The dependent variable in: Regression 1 uses the upper and lower bounds of participants' implied risk-aversion coefficients from the B–EG task. Regression 2 uses a 6-point scale from the adapted B–EG task that includes losses, ranging from 1 = most risk-seeking (Lottery F) to 6 = most risk-averse (Lottery A). Regressions 3 and 4 use a dichotomous indicator equal to 1 for loss-free lotteries (Lotteries A–C) and 0 for lotteries that include a loss (Lotteries D–F) from the adapted B–EG task. Polygenic score for IQ (PGS IQ) is standardized. Unadjusted coefficients; z-statistics using robust standard errors in brackets. Controls include age (in linear and quadratic form), sex, and the first 10 principal components of respondents' SNPs. In Regression 4, we also include a series of dummy variables representing respondents' choice in the B-EG task. In all regressions, all parameters are allowed to differ across groups ('no disadvantage' and 'disadvantage').

* p<0.10, ** p<0.05, *** p<0.01.

Supplementary Table 19. Gene-Environment Interaction and Time Preferences – PGS IQ

Regression:	1	2	3	4	5
Dependent variable:	Discount rate (1-Month MPL)	Discount rate (2-Month MPL)	Discount rate (2-Month MPL)	Planning horizon	Planning horizon (binary)
Estimator:	Interval	Interval	Interval	Ordered Logit	Logit
$\beta 1$ PGS IQ	-0.020**	-0.005*	0.000	0.137***	0.150***
	[-2.421]	[-1.778]	[0.149]	[5.677]	[5.570]
$\beta 2$ PGS IQ × Disadv.	0.018	0.005	0.000	-0.082**	-0.092**
	[1.326]	[1.121]	[0.139]	[-2.078]	[-2.107]
$\beta 1 + \beta 2$	-0.002	0.000	0.001	0.055*	0.058*
	[-0.218]	[0.075]	[0.313]	[1.746]	[1.693]
Controls	Yes	Yes	Yes	Yes	Yes
Observations	624	624	624	11521	11521

Note. The dependent variable in: Regression 1 uses the upper and lower bounds of participants' implied discount rate from the 1-month multiple price list (MPL) task. Regressions 2 and 3 use participants' implied discount rates from the 2-month MPL task. Regression 4 uses a 7-point planning horizon scale ranging from 0 ("does not plan/plans day-to-day") to 6 ("longer than 10 years"). Regression 5 uses a dichotomous planning horizon indicator equal to 1 if the respondent reports planning beyond the next year, and 0 otherwise. Polygenic score for IQ (PGS IQ) is standardized. Unadjusted coefficients; z-statistics using robust standard errors in brackets (cluster-robust standard errors in Regressions 4 and 5). Controls include age (in linear and quadratic form), sex, and the first 10 principal components of respondents' SNPs. In Regressions 1-3 we also include a dichotomous control variable that equals 1 if the subject made an inconsistent choice in the MPL task and 0 otherwise. In Regression 3, we also include a series of dummy variables representing respondents' choice from the 1-month MPL task. In all regressions, all parameters are allowed to differ across groups ('no disadvantage' and 'disadvantage').
* p<0.10, ** p<0.05, *** p<0.01.

Supplementary Table 20. Phenotypic Cognitive Ability-Environment Interaction and Risk Preferences

Regression:	1	2	3	4
Dependent variable:	Risk aversion (B-EG)	Risk aversion (LA)	Risk aversion (LA-binary)	Risk aversion (LA-binary)
Estimator:	Interval	Ordered Logit	Logit	Logit
$\beta1$ Phenotypic cognitive ability	-0.757***	-0.428***	-0.131	0.079
	[-3.064]	[-2.703]	[-0.636]	[0.339]
$\beta2$ Phenotypic cognitive ability × Disadv.	1.063***	0.801***	0.687**	0.397
	[2.873]	[2.962]	[2.199]	[1.157]
$\beta1 + \beta2$	0.305	0.373*	0.556**	0.476*
	[1.108]	[1.704]	[2.364]	[1.886]
Controls	Yes	Yes	Yes	Yes
Observations	624	624	624	624

Note. The dependent variable in: Regression 1 uses the upper and lower bounds of participants' implied risk-aversion coefficients from the B–EG task. Regression 2 uses a 6-point scale from the adapted B–EG task that includes losses, ranging from 1 = most risk-seeking (Lottery F) to 6 = most risk-averse (Lottery A). Regressions 3 and 4 use a dichotomous indicator equal to 1 for loss-free lotteries (Lotteries A–C) and 0 for lotteries that include a loss (Lotteries D–F) from the adapted B–EG task. Phenotypic cognitive ability is standardized. Unadjusted coefficients; z-statistics using robust standard errors in brackets. Controls include age (in linear and quadratic form) and sex. In Regression 4, we also include a series of dummy variables representing respondents' choice in the B-EG task. In all regressions, all parameters are allowed to differ across groups ('no disadvantage' and 'disadvantage').
* p<0.10, ** p<0.05, *** p<0.01.

Supplementary Table 21. Phenotypic Cognitive Ability-Environment Interaction and Time Preferences

Regression:	1	2	3	4	5
Dependent variable:	Discount rate (1-Month MPL)	Discount rate (2-Month MPL)	Discount rate (2-Month MPL)	Planning horizon	Planning horizon (binary)
Estimator:	Interval	Interval	Interval	Ordered Logit	Logit
$\beta 1$ Phenotypic cognitive ability	-0.059***	-0.021***	-0.005	0.509***	0.527***
	[-3.809]	[-3.664]	[-1.081]	[14.771]	[13.147]
$\beta 2$ Phenotypic cognitive ability IQ × Disadv.	-0.006	-0.002	-0.000	-0.207***	-0.275***
	[-0.256]	[-0.266]	[-0.017]	[-3.476]	[-4.227]
$\beta 1 + \beta 2$	-0.065***	-0.023***	-0.005	0.302***	0.251***
	[-3.901]	[-3.453]	[-1.080]	[6.168]	[4.885]
Controls	Yes	Yes	Yes	Yes	Yes
Observations	624	624	624	11521	11521

Note. The dependent variable in: Regression 1 uses the upper and lower bounds of participants' implied discount rate from the 1-month multiple price list (MPL) task. Regressions 2 and 3 use participants' implied discount rates from the 2-month MPL task. Regression 4 uses a 7-point planning horizon scale ranging from 0 ("does not plan/plans day-to-day") to 6 ("longer than 10 years"). Regression 5 uses a dichotomous planning horizon indicator equal to 1 if the respondent reports planning beyond the next year, and 0 otherwise. Phenotypic cognitive ability is standardized. Unadjusted coefficients; z-statistics using robust standard errors in brackets (cluster-robust standard errors in Regressions 4 and 5). Controls include age (in linear and quadratic form) and sex. In Regressions 1-3 we also include a dichotomous control variable that equals 1 if the subject made an inconsistent choice in the MPL task and 0 otherwise. In Regression 3, we also include a series of dummy variables representing respondents' choice from the 1-month MPL task. In all regressions, all parameters are allowed to differ across groups ('no disadvantage' and 'disadvantage').

* p<0.10, ** p<0.05, *** p<0.01.

Supplementary Table 22. Gene-Environment Interaction and Risk Preferences – Including Wealth, Income, and Educational Attainment

Regression:	1	2	3	4
Dependent variable:	Risk aversion (B-EG)	Risk aversion (LA)	Risk aversion (LA-binary)	Risk aversion (LA-binary)
Estimator:	Interval	Ordered Logit	Logit	Logit
$\beta 1$ PGS EA	-0.358***	-0.340***	-0.599***	-0.541***
	[-2.737]	[-3.678]	[-4.783]	[-4.087]
$\beta 2$ PGS EA × Disadv.	0.826***	0.713***	0.755***	0.635**
	[3.619]	[3.952]	[3.372]	[2.570]
$\beta 1 + \beta 2$	0.468**	0.373**	0.156	0.093
	[2.497]	[2.417]	[0.841]	[0.448]
Controls	Yes	Yes	Yes	Yes
Observations	624	624	624	624

Note. The dependent variable in: Regression 1 uses the upper and lower bounds of participants' implied risk-aversion coefficients from the B–EG task. Regression 2 uses a 6-point scale from the adapted B–EG task that includes losses, ranging from 1 = most risk-seeking (Lottery F) to 6 = most risk-averse (Lottery A). Regressions 3 and 4 use a dichotomous indicator equal to 1 for loss-free lotteries (Lotteries A–C) and 0 for lotteries that include a loss (Lotteries D–F) from the adapted B–EG task. Polygenic score for educational attainment (PGS EA) is standardized. Unadjusted coefficients; z-statistics using robust standard errors in brackets. Controls include age (in linear and quadratic form), sex, the first 10 principal components of respondents' SNPs, logarithm of net total household wealth (the sum of savings, investments, physical wealth, and housing wealth after financial debt and mortgage debt has been subtracted), logarithm of annual household total income, and the highest reported level of educational attainment. In Regression 4, we also include a series of dummy variables representing respondents' choice in the B-EG task. In all regressions, all parameters are allowed to differ across groups ('no disadvantage' and 'disadvantage').

* p<0.10, ** p<0.05, *** p<0.01.

Supplementary Table 23. Gene-Environment Interaction and Time Preferences – Including Wealth, Income, and Educational Attainment

Regression:	1	2	3	4	5
Dependent variable:	Discount rate (1-Month MPL)	Discount rate (2-Month MPL)	Discount rate (2-Month MPL)	Planning horizon	Planning horizon (binary)
Estimator:	Interval	Interval	Interval	Ordered Logit	Logit
$\beta 1$ PGS EA	-0.012	-0.009***	-0.006**	0.059**	0.058**
	[-1.429]	[-2.895]	[-2.450]	[2.288]	[2.005]
$\beta 2$ PGS EA × Disadv.	-0.009	0.008	0.011***	-0.077*	-0.100**
	[-0.671]	[1.490]	[2.792]	[-1.860]	[-2.123]
$\beta 1 + \beta 2$	-0.022**	-0.002	0.005*	-0.018	-0.042
	[-1.991]	[-0.372]	[1.656]	[-0.546]	[-1.132]
Controls	Yes	Yes	Yes	Yes	Yes
Observations	624	624	624	11472	11472

Note. The dependent variable in: Regression 1 uses the upper and lower bounds of participants' implied discount rate from the 1-month multiple price list (MPL) task. Regressions 2 and 3 use participants' implied discount rates from the 2-month MPL task. Regression 4 uses a 7-point planning horizon scale ranging from 0 ("does not plan/plans day-to-day") to 6 ("longer than 10 years"). Regression 5 uses a dichotomous planning horizon indicator equal to 1 if the respondent reports planning beyond the next year, and 0 otherwise. Polygenic score for educational attainment (PGS EA) is standardized. Unadjusted coefficients; z-statistics using robust standard errors in brackets (cluster-robust standard errors in Regressions 4 and 5). Controls include age (in linear and quadratic form), sex, the first 10 principal components of respondents' SNPs, logarithm of net total household wealth (the sum of savings, investments, physical wealth, and housing wealth after financial debt and mortgage debt has been subtracted), logarithm of annual household total income, and the highest reported level of educational attainment. In Regressions 1-3 we also include a dichotomous control variable that equals 1 if the subject made an inconsistent choice in the MPL task and 0 otherwise. In Regression 3, we also include a series of dummy variables representing respondents' choice from the 1-month MPL task. In all regressions, all parameters are allowed to differ across groups ('no disadvantage' and 'disadvantage').
* p<0.10, ** p<0.05, *** p<0.01.

Supplementary Note 1: Phenotypic Cognitive Ability

The English Longitudinal Study of Ageing (ELSA) includes a wide array of tasks aimed at evaluating various aspects of cognitive functioning, such as memory, executive processing, and fundamental cognitive capabilities.

The first task focused on temporal orientation—conducted in all waves—where respondents were asked to report the current day, month, year, and day of the week. The final score is based on a simple count of number of correct items.

The second task involved a word recall assessment designed to measure verbal memory. Participants in all Waves were read a list of 10 words and immediately afterwards were asked to recall as many words as possible, in any order. They were then prompted again, later in the interview, to recall as many words as possible, in any order. The total number of correctly recalled words across both the immediate and delayed implementation were combined into a single score.

A third task assessed prospective memory and was conducted during Waves 1 to 5 and again in Wave 8. Participants were given two tasks to remember: one involved—when handed a clipboard and a pencil by the interviewer—writing their initials on the top left-hand corner of piece of paper attached to the clipboard, and another required them to remind the interviewer to record the time at the end of the session. Performance was evaluated based on task completion and the need for prompts.

Numeracy skills were assessed through a fourth task, administered in Waves 1, 4, and to refreshment samples in Waves 6 to 9. This task involved solving up to five arithmetic problems of increasing difficulty, depending on performance on initial questions. Specifically, based on performance on the first three items, participants can get two additional (more difficult) questions or one additional (simpler) question. Sample items included estimating the original cost of a car sold at two-thirds of its price and calculating an equal lottery payout. Scoring reflected the number of accurate responses.

Executive function was examined through a counting backwards task (Wave 7 to 9), which asked participants to count backwards quickly from 20. Scoring was binary: either participants counted correctly from 19 to 10 or from 20 to 11, or they did not.

Executive function was also assessed through an object-naming assessment, conducted in Waves 7 to 9, where participants were required to name five common objects or people in response to prompts. The type of questions asked included: "*What do you call the kind of*

prickly plant that grows in the desert?" and "*Who is the reigning monarch now?*". Scores reflected the number of correct identifications.

A Serial 7s subtraction task (also in Waves 7 to 9) tested numerical working memory and attention by asking respondents to sequentially subtract 7 from 100, up to five times. The final score captured the number of correct subtractions.

Semantic verbal fluency was tested using an animal-naming task (all waves except Wave 6), where participants had one minute to list as many animals as possible. The score represented the count of unique, valid responses.

A further task measuring processing speed and accuracy was a letter cancellation task (Waves 1 to 5), in which participants scanned lines of letters and marked all instances of the letter's "P" and "W." Participants were asked to start at the top left-hand corner of the list and work along each line of the list sequentially, as if they were reading a page. Participants were asked to work as quickly and as accurately as they could, until the computer says stop. At this point, participants were asked to underline the last letter on which they finished. Two scores were generated: one for the number of P's and W's correctly marked and another for the number of missed letters up to the marked stopping point.

A fluid intelligence task was added in Waves 6, 8, and 9 using a number series exercise. Respondents were presented with number sequences containing a missing element and asked to identify the correct missing number. Based on their performance in an initial set of three sequences, they received a second set of problems, with difficulty tailored to their earlier performance. Scoring incorporated both accuracy and problem difficulty.

Finally, literacy was assessed through a reading comprehension task included in Waves 2 and 5, and with refreshment samples in Waves 6 to 9. Participants were asked to read a medication label and answer four comprehension questions, such as identifying usage restrictions or appropriate conditions for consulting a doctor. The final score counted the number of correct answers.

A composite measure representing general cognitive ability was constructed from twelve individual cognitive test scores. This was calculated by standardizing and combining respondents' average scores—as individuals may take each cognitive task more than once—across the tasks. The general cognitive ability score includes all available non-missing items for each individual. As a result, observations were retained as long as at least one component variable was observed, rather than being dropped due to any single missing value. The resulting composite demonstrated acceptable internal reliability, with a Cronbach's alpha of 0.78 and an average inter-item correlation of 0.23.

Supplementary Note 2: Validating the Polygenic Score for Educational Attainment (PGS EA)

To validate the polygenic score for educational attainment (PGS EA) we formally estimate its relationship with two cognitive phenotypes: educational attainment and cognitive ability. Educational attainment is measured as a binary variable coded 1 for respondents who reported a university or college degree, and 0 for all other qualification levels (including those with no formal qualification). Cognitive ability is measured, following Dawson (2015), as a general IQ factor from a range of cognitive function tasks—designed to measure memory, executive function, and basic cognitive skills/abilities—with acceptable-to-good internal consistency reliability across the items. For a full description of the cognitive tasks, see Supplementary Note 1. In addition to establishing the association of PGS EA with phenotypic educational attainment and cognitive ability, we test the Scarr–Rowe hypothesis: that cognitive-related genetic associations are more fully realized in supportive, resource-rich settings (Rowe et al., 1999; Scarr-Salapatek, 1971; Tucker-Drob et al., 2013). In our analysis we use the standard between-family model for gene-by-environment interactions (Papageorge & Thom, 2020; Ronda et al., 2022), described as:

$$P_{ig}^{k} = \alpha^{k}(g)PGS_{ig}^{EA} + X_{ig}b^{k}(g) + \epsilon_{ig}^{k} \qquad (1)$$

where P_{ig}^{k} is the kth phenotypic outcome (i.e., educational attainment or cognitive ability) for individual i in group g (i.e., 'no disadvantage' or 'disadvantage'), PGS_{ig}^{EA} is the polygenic score for educational attainment, and X_{ig} is a vector of exogenous control variables including year of birth, sex, and the first 10 principal components of the respondent's SNPs—which allows us to control for any ancestry differences in genetic structures. For instance, if a particular SNP variant is more common in a specific ancestry group, an observed association between PGS^{EA} and phenotypic outcomes may reflect cultural norms shared by this ancestry group. Lastly, ϵ_{ig}^{k} is the usual random error component. This approach allows all parameters to differ across g, with our primary interest being the estimated genetic effects, α^{k} and the comparison of the estimated genetic effects by group, g.

The results are presented in Supplementary Table 24 (Supplementary Figure 1), where $\beta 1$ ($\beta 1 + \beta 2$) is an estimate of the effect of the PGS EA on the dependent variable for the 'no disadvantage' ('disadvantage') group and $\beta 2$ is the comparison of the estimated genetic effects, $\alpha^{k}(g)$, between the 'no disadvantage' and 'disadvantage' groups.

The results show that the educational-attainment polygenic score (PGS EA) is a strong predictor of completing a university or college degree. However, in contrast to the Scarr–Rowe pattern, we do not find evidence that this predictive power varies systematically by childhood socioeconomic conditions. For both the experimental and survey samples, the PGS EA predicts educational attainment to a similar extent among individuals raised in advantaged and disadvantaged environments.

For cognitive ability, the predictive effect of the PGS EA is likewise independent of childhood background, consistent with prior research showing that environmental moderation of cognitive heritability diminishes or disappears in adulthood (Gottschling et al., 2019; Tucker-Drob & Bates, 2016). This accords with the broader developmental literature suggesting that the influence of shared early environments on individual differences declines over the life course (Briley & Tucker-Drob, 2013; Plomin & Daniels, 1987; Plomin & von Stumm, 2018; Segal & Pratt-Thompson, 2024; Turkheimer, 2000). Although the interaction is absent, individuals who experienced childhood disadvantage exhibit lower predicted probabilities of completing a university or college degree—and lower predicted cognitive ability—at every level of the PGS EA distribution. Supplementary Figure 1 illustrates these relationships by plotting the predicted probability of obtaining a university or college degree (and predicted cognitive ability) across the PGS EA distribution for both childhood-environment groups.

The absence of Scarr–Rowe–type moderation for either educational attainment or cognitive ability indicates that disadvantaged childhood environments do not suppress the expression of genetic cognitive propensities in our sample. Although cognitive skills are widely viewed as a key input into economic preferences, this pattern suggests that any variation in how polygenic influences relate to preferences across childhood contexts is unlikely to arise from differences in the expression of cognitive-related genetic variation.

Supplementary Table 24. Gene-Environment Interaction and Cognitive Phenotypes

Regression:	1	2	3	4
Dependent variable:	University or college degree	Cognitive ability	University or college degree	Cognitive ability
Sample:	Experimental		Survey	
Estimator:	Logit	OLS	Logit	OLS
$\beta 1$ PGS EA	0.576***	0.110***	0.591***	0.138***
	[5.049]	[4.215]	[18.279]	[17.273]
$\beta 2$ PGS EA × Disadv.	-0.111	0.020	0.017	-0.002
	[-0.394]	[0.398]	[0.260]	[-0.145]
$\beta 1 + \beta 2$	0.465*	0.131***	.608***	0.136***
	[1.800]	[2.953]	[10.746]	[11.608]
Controls	Yes	Yes	Yes	Yes
Observations	624	624	11521	11521

Note. The dependent variable in: Regressions 1 and 3 uses a dichotomous variable coded 1 for respondents who reported a university or college degree, and 0 for all other qualification levels (including those with no formal qualification). Regressions 2 and 4 uses a general cognitive factor from a barrage of cognitive tests designed to measure memory, executive function, and basic cognitive skills/abilities. Polygenic score for educational attainment (PGS EA) and phenotypic cognitive ability are standardized. Unadjusted coefficients; z-statistics using robust standard errors in brackets (cluster-robust standard errors in Regressions 3 and 4). Controls include date of birth, sex, and the first 10 principal components of respondents' SNPs. In all regressions, all parameters are allowed to differ across groups ('no disadvantage' and 'disadvantage').

* p<0.10, ** p<0.05, *** p<0.01.

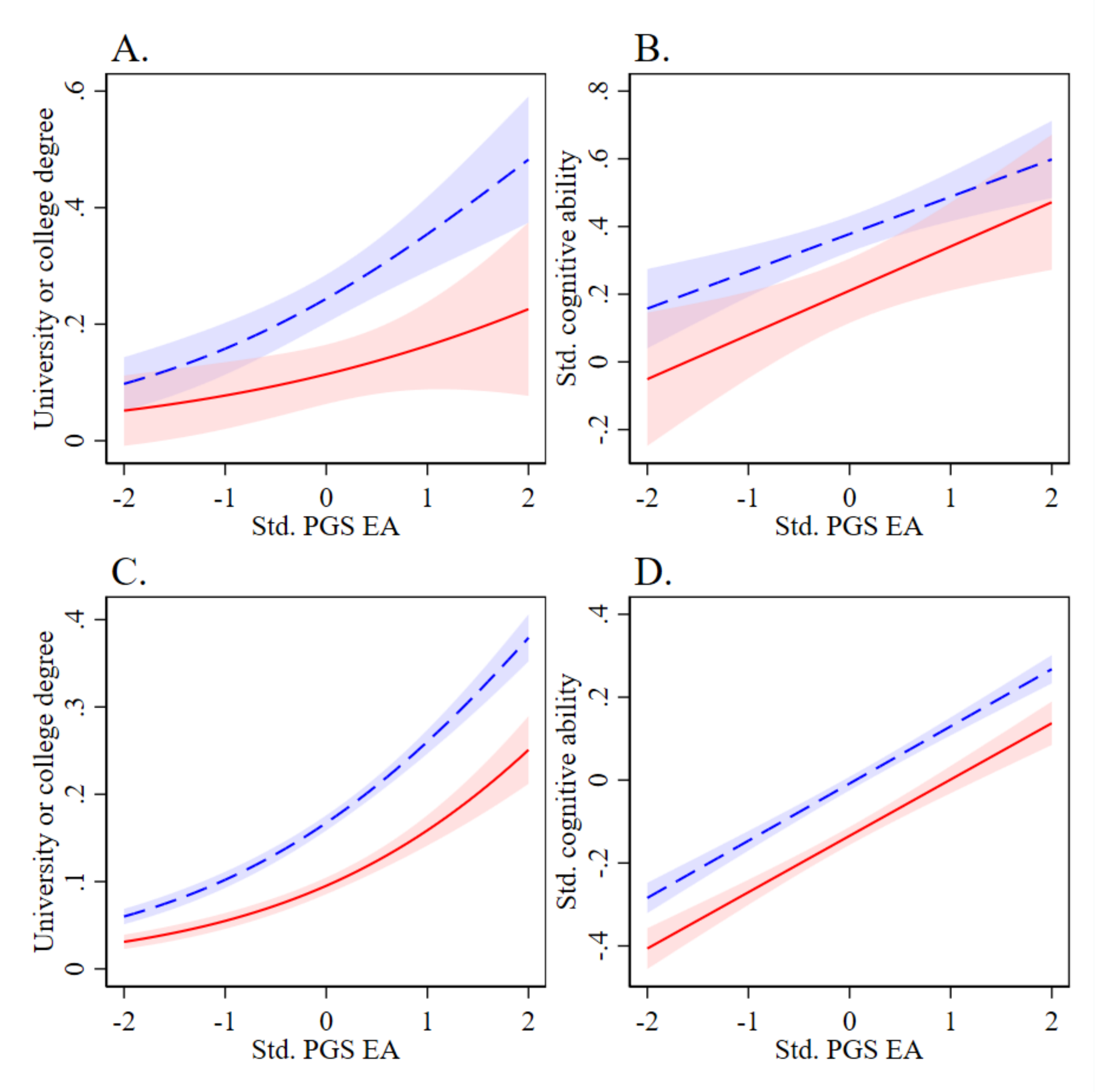

Supplementary Figure 1. Overview of time-preference patterns by PGS EA and childhood disadvantage.

Figure legend.

Panels A–D show predicted levels/probabilities of cognitive phenotypes across the range of standardized polygenic scores for educational attainment (PGS EA). In Panels A and B, predictions are based on the experimental sample (*N* = 624); in Panels C and D, predictions are based on the survey sample (*N* = 5,881; 11,521 person-wave observations). The red solid line represents respondents who experienced childhood disadvantage, and the blue dashed line represents respondents without childhood disadvantage; light red and light blue shaded regions depict 95% confidence intervals. Panels A and C plot the predicted probability of reporting a university or college degree vs. all other qualification levels (including those with no formal qualification). Panels B and D plot predicted phenotypic cognitive ability, based on a battery of cognitive tests designed to measure memory, executive function, and general cognitive skills/abilities. All predictions control for age (in linear and quadratic form), sex, and the first 10 principal components of respondents' SNPs. Standardized PGS EA is plotted on the horizontal axis in all panels.

Supplementary References

Briley, D. A., & Tucker-Drob, E. M. (2013). Explaining the increasing heritability of cognitive ability across development: A meta-analysis of longitudinal twin and adoption studies. *Psychological Science*, *24*(9), 1704-1713.

Dawson, C. (2025). IQ, Genes, and Miscalibrated Expectations. *Journal of Personality and Social Psychology*. Advance online publication. https://doi.org/10.1037/pspp0000567

de Moor, M. H., Costa, P. T., Terracciano, A., Krueger, R. F., De Geus, E. J., Toshiko, T., ... & Boomsma, D. I. (2012). Meta-analysis of genome-wide association studies for personality. Molecular Psychiatry, 17(3), 337-349.

Gottschling, J., Hahn, E., Beam, C. R., Spinath, F. M., Carroll, S., & Turkheimer, E. (2019). Socioeconomic status amplifies genetic effects in middle childhood in a large German twin sample. *Intelligence*, *72*, 20-27.

Okbay, A., Baselmans, B. M., De Neve, J. E., Turley, P., Nivard, M. G., Fontana, M. A., ... & Power, C. (2016). Genetic variants associated with subjective well-being, depressive symptoms, and neuroticism identified through genome-wide analyses. Nature Genetics, 48(6), 624-633.

Papageorge, N. W., & Thom, K. (2020). Genes, education, and labor market outcomes: evidence from the health and retirement study. *Journal of the European Economic Association*, *18*(3), 1351-1399.

Plomin, R., & Daniels, D. (1987). Why are children in the same family so different from one another? *Behavioral and Brain Sciences*, *10*(1), 1-16.

Plomin, R., & von Stumm, S. (2018). The new genetics of intelligence. *Nature Reviews Genetics*, *19*(3), 148-159.

Ronda, V., Agerbo, E., Bleses, D., Mortensen, P. B., Børglum, A., Mors, O., ... & Werge, T. (2022). Family disadvantage, gender, and the returns to genetic human capital. *The Scandinavian Journal of Economics*, *124*(2), 550-578.

Rowe, D. C., Jacobson, K. C., & Van den Oord, E. J. (1999). Genetic and environmental influences on vocabulary IQ: Parental education level as moderator. *Child Development*, 70(5), 1151–1162.

Scarr-Salapatek, S. (1971). Race, Social Class, and IQ: Population differences in heritability of IQ scores were found for racial and social class groups. *Science*, *174*(4016), 1285-1295.

Segal, N. L., & Pratt-Thompson, E. (2024). Developmental trends in intelligence revisited with novel kinships: Monozygotic twins reared apart v. same-age unrelated siblings reared together. *Personality and Individual Differences*, *229*, 112751.

Tucker-Drob, E. M., Briley, D. A., & Harden, K. P. (2013). Genetic and environmental influences on cognition across development and context. *Current Directions in Psychological Science*, *22*(5), 349-355.

Tucker-Drob, E. M., & Bates, T. C. (2016). Large cross-national differences in gene× socioeconomic status interaction on intelligence. *Psychological Science*, *27*(2), 138-149.

Turkheimer, E. (2000). Three laws of behavior genetics and what they mean. *Current Directions in Psychological Science*, *9*(5), 160-164.

van den Berg, S. M., de Moor, M. H., Verweij, K. J., Krueger, R. F., Luciano, M., Arias Vasquez, A., ... & Boomsma, D. I. (2016). Meta-analysis of genome-wide association studies for extraversion: findings from the genetics of personality consortium. *Behavior Genetics*, *46*, 170-182.
