# Supplementary material for: Associations of genetic variants for educational success with risk and time preferences vary by childhood environment
Source: Commun Psychol. 2026 Feb 11;4:50. doi: 10.1038/s44271-026-00421-y (PMC13004898; doi:10.1038/s44271-026-00421-y)
Supplement: Supplementary file 3 — Reporting Summary [file 44271_2026_421_MOESM3_ESM.pdf]

nature portfolio

Corresponding author(s): COMMSPSYCHOL-25-0734-T

Last updated by author(s): Jan 26, 2026

Reporting Summary

Nature Portfolio wishes to improve the reproducibility of the work that we publish. This form provides structure for consistency and transparency in reporting. For further information on Nature Portfolio policies, see our Editorial Policies and the Editorial Policy Checklist.

Statistics

For all statistical analyses, confirm that the following items are present in the figure legend, table legend, main text, or Methods section.

n/a	Confirmed	
☐	☒	The exact sample size (n) for each experimental group/condition, given as a discrete number and unit of measurement
☐	☒	A statement on whether measurements were taken from distinct samples or whether the same sample was measured repeatedly
☒	☐	The statistical test(s) used AND whether they are one- or two-sided<br>*Only common tests should be described solely by name; describe more complex techniques in the Methods section.*
☐	☒	A description of all covariates tested
☐	☒	A description of any assumptions or corrections, such as tests of normality and adjustment for multiple comparisons
☐	☒	A full description of the statistical parameters including central tendency (e.g. means) or other basic estimates (e.g. regression coefficient) AND variation (e.g. standard deviation) or associated estimates of uncertainty (e.g. confidence intervals)
☐	☒	For null hypothesis testing, the test statistic (e.g. F, t, r) with confidence intervals, effect sizes, degrees of freedom and P value noted<br>*Give P values as exact values whenever suitable.*
☒	☐	For Bayesian analysis, information on the choice of priors and Markov chain Monte Carlo settings
☐	☒	For hierarchical and complex designs, identification of the appropriate level for tests and full reporting of outcomes
☒	☐	Estimates of effect sizes (e.g. Cohen's d, Pearson's r), indicating how they were calculated

Our web collection on statistics for biologists contains articles on many of the points above.

Software and code

Policy information about availability of computer code

Data collection

Data come from the English Longitudinal Study of Ageing (ELSA), 1998–2023 (Waves 0–10), a representative panel of adults aged 50 and over in England. The experimental sample includes 624 individuals (aged 50–75, mean = 64.18, SD = 5.61), and the survey sample includes 5,881 individuals contributing 11,521 observations (aged 31–90, mean = 64.46, SD = 9.43). Sex was self-reported by participants in response to a prompt asking for biological sex with response options Female or Male. In the experimental sample, 335 participants (53.7%) reported female, and 289 participants (46.3%) reported male. In the survey sample, 3,209 participants (54.6%) reported female, and 2,672 participants (45.4%) reported male. Genetic analyses were restricted to participants of European ancestry, consistent with polygenic score derivation. Childhood and adult socioeconomic information, including parental human capital, material resources, and household stability, were collected and included in analyses.All participants provided informed consent at the time of their interview, including additional consent for biomarker and genetic data collection where applicable. Experimental module participants received a £10 participation fee and additional real monetary payoffs determined by their choices.

Data analysis

The data was analyzed using Stata. We provide the following Open Practices Statement. The data that support the findings of this study are publicly available from the UK Data Archive. The complete Stata analysis script to replicate the results is openly available in Open Science Framework (OSF) at: osf.io/f8teh

For manuscripts utilizing custom algorithms or software that are central to the research but not yet described in published literature, software must be made available to editors and reviewers. We strongly encourage code deposition in a community repository (e.g. GitHub). See the Nature Portfolio guidelines for submitting code & software for further information.

Data

Policy information about availability of data

All manuscripts must include a data availability statement. This statement should provide the following information, where applicable:

- Accession codes, unique identifiers, or web links for publicly available datasets
- A description of any restrictions on data availability
- For clinical datasets or third party data, please ensure that the statement adheres to our policy

Open Practices Statement. The data that support the findings of this study are publicly available from the UK Data Archive. The complete STATA analysis script to replicate the results is openly available in Open Science Framework (OSF) at: osf.io/f8teh

Research involving human participants, their data, or biological material

Policy information about studies with human participants or human data. See also policy information about sex, gender (identity/presentation), and sexual orientation and race, ethnicity and racism.

Reporting on sex and gender	Biological sex is include as a covariate.
Reporting on race, ethnicity, or other socially relevant groupings	No data on race was used as the study uses genetic data which is restricted to participants of European ancestry, consistent with polygenic score derivation.
Population characteristics	The data is secondary, and represents a national representative survey of people from the UK.
Recruitment	Secondary national representative data: https://www.elsa-project.ac.uk/accessing-elsa-data
Ethics oversight	This study is based on the analysis of anonymized secondary data from the English Longitudinal Study of Ageing (ELSA) available from the UK Data Service. Information on the ethical approval received for each wave of ELSA can be found at: https://www.elsa-project.ac.uk/ethical-approval. This study (application reference number: 10956-12470) received a favorable opinion through the University of Bath's Social Science Research Ethics Committee's review process.

Note that full information on the approval of the study protocol must also be provided in the manuscript.

Field-specific reporting

Please select the one below that is the best fit for your research. If you are not sure, read the appropriate sections before making your selection.

☐ Life sciences ☒ Behavioural & social sciences ☐ Ecological, evolutionary & environmental sciences

For a reference copy of the document with all sections, see nature.com/documents/nr-reporting-summary-flat.pdf

Behavioural & social sciences study design

All studies must disclose on these points even when the disclosure is negative.

Study description	Quantitative secondary data
Research sample	Nationally representative sample of UK respondents
Sampling strategy	ELSA describes its collection of data on its website: https://www.elsa-project.ac.uk/about-elsa
Data collection	See above
Timing	The original sample was drawn from households that had previously responded to the Health Survey for England (HSE) between 1998 and 2001. A pilot study was conducted in 2001 before main fieldwork began in March 2002. The same group of respondents have been interviewed at two-yearly interviews, known as 'waves', to measure changes in their health, economic and social circumstances. Younger age groups are replaced or refreshed to retain the panel. The sample has been refreshed using HSE participants in waves 3, 4, 6, 7, 9, and 10.
Data exclusions	No data restrictions were imposed other than on the availability of variables
Non-participation	ELSA describes its collection of data on its website: https://www.elsa-project.ac.uk/about-elsa
Randomization	ELSA describes its collection of data on its website: https://www.elsa-project.ac.uk/about-elsa

Reporting for specific materials, systems and methods

We require information from authors about some types of materials, experimental systems and methods used in many studies. Here, indicate whether each material, system or method listed is relevant to your study. If you are not sure if a list item applies to your research, read the appropriate section before selecting a response.

Materials & experimental systems

n/a	Involved in the study
☒	☐ Antibodies
☒	☐ Eukaryotic cell lines
☒	☐ Palaeontology and archaeology
☒	☐ Animals and other organisms
☒	☐ Clinical data
☒	☐ Dual use research of concern
☒	☐ Plants

Methods

n/a	Involved in the study
☒	☐ ChIP-seq
☒	☐ Flow cytometry
☒	☐ MRI-based neuroimaging

Plants

Seed stocks	*Report on the source of all seed stocks or other plant material used. If applicable, state the seed stock centre and catalogue number. If plant specimens were collected from the field, describe the collection location, date and sampling procedures.*
Novel plant genotypes	*Describe the methods by which all novel plant genotypes were produced. This includes those generated by transgenic approaches, gene editing, chemical/radiation-based mutagenesis and hybridization. For transgenic lines, describe the transformation method, the number of independent lines analyzed and the generation upon which experiments were performed. For gene-edited lines, describe the editor used, the endogenous sequence targeted for editing, the targeting guide RNA sequence (if applicable) and how the editor was applied.*
Authentication	*Describe any authentication procedures for each seed stock used or novel genotype generated. Describe any experiments used to assess the effect of a mutation and, where applicable, how potential secondary effects (e.g. second site T-DNA insertions, mosiacism, off-target gene editing) were examined.*
